# Anti-FIM and Anti-FHA Antibodies Inhibit *Bordetella pertussis* Growth and Reduce Epithelial Cell Inflammation Through Bacterial Aggregation

**DOI:** 10.3389/fimmu.2020.605273

**Published:** 2020-12-15

**Authors:** Issaka Yougbare, Adam McTague, Liwei He, Christopher H. Choy, Jin Su, Beata Gajewska, Ali Azizi

**Affiliations:** Immunology platform, Analytical Sciences North America, Sanofi Pasteur, Toronto, ON, Canada

**Keywords:** anti-pertussis antibodies, aggregation, bacterial growth inhibition, epithelial inflammation, cytokines

## Abstract

The pertussis vaccination is highly recommended for infants, children, and pregnant women. Despite a high coverage of vaccination, pertussis continues to be of public health concern as a re-emerging infectious disease. The mechanism by which vaccine-elicited anti-pertussis antibodies mediate direct bactericidal effects is poorly understood. In this study, we showed that the interaction of *B. pertussis* with A549 epithelial cells induce release of biological factors which enhance bacteria growth. Complement-depleted antisera from vaccine-immunized guinea pigs or monoclonal antibodies targeting FHA and FIM mediate bacteria aggregation and elicit bactericidal effects. Our *in vitro* results indicated that aggregation of bacteria through anti-FIM and anti-FHA specific antibodies is one of the major biological mechanisms to clear bacterial infections and restore epithelial cell survival *in vitro*. Our data also indicates that the anti-pertussis antibodies reduce secretion of proinflammatory chemokines and cytokines by preventing interaction of *B. pertussis* with host cells. The results of this study not only demonstrate mechanism of action of anti-FIM and anti-FHA antibodies, but also opens translational applications for potential therapeutic approaches or development of analytical assays such as *in vitro* potency assays.

## Introduction

Pertussis, also known as whooping cough, is a re-emerging infectious disease in many countries despite high coverage of vaccines (1, 2). This life-threatening contagious disease is caused by the Gram-negative bacterium, *Bordetella pertussis*. Re-emergence of pertussis despite widespread vaccination is due to various parameters including the selection or evolution of mutant *B. pertussis* strains or waning protection of acellular vaccines (1, 3–5). For an effective control of pertussis, it is recommended to strengthen vaccination coverage among the whole population by providing primary vaccination to newborns, and boosting infants and adults every 10 years (6). The recent re-emergence of the disease in North America showed that pertussis is particularly dangerous for infants under 4 months of age, accounting for 86% of all pertussis associated fatalities (7). Vaccination of women at the third trimester of pregnancy has been shown to be the most effective intervention to protect the newborn from pertussis (8). The passively transferred maternal antibodies to the neonates play a protective role before pertussis vaccination can be implemented (9, 10). Studies have shown strong humoral protection in infants; however, the mechanism by which antibodies control *B. pertussis* infection has not been well understood (9, 11, 12).

Adverse reactions after immunization with the whole-cell pertussis (wP) vaccine led to the development of acellular pertussis (aP) vaccines with less reactogenicity (13). The aP-based vaccines contain up to five pertussis antigens including pertactin (PRN), filamentous hemagglutinin adhesin (FHA), fimbriae 2 and 3 (FIM), and pertussis toxoid (PT). Among several adhesion proteins produced by *B. pertussis*, FIM, and FHA have been shown to allow the bacterium to attach to host epithelial and immune cells. Agglutinogens FIM2 and FIM3 are key adhesion proteins with the molecular weight 22 kDa and 22.5 kDa respectively. While FIM2 and FIM 3 are structurally related, they are serologically distinct (14). It has been suggested that FIM-mediated adherence of *B. pertussis* to airway epithelium is a first step in infection, allowing adherence, suppressing inflammation, and enhancing resistance to inflammatory cell-mediated clearance (15). FHA is a 220 kDa surface-associated protein that predominantly serves as an adhesion molecule to host ciliated epithelial cells, therefore FHA is associated with biofilm formation, and persistence of the infection (16–18). FHA possesses four binding domains which can bind to different cell receptors on the epithelial cell surface; its immune modulatory effects have also been reported on immune cells (19). PT and PRN have been also reported as adhesins, however, their roles in *B. pertussis* adhesion to host cells remain controversial (20, 21).

The mechanism underlining antibody mediated inhibition of bacterial adhesion to the epithelium and any potential bactericidal effect is poorly understood. The aim of this study was to explore the function of pertussis adhesion antigens (FHA and FIM) by inhibiting their interactions with host epithelial cells (A549) using antigen-specific antibodies. To achieve this, we have established a co-culture system with *B. pertussis* and A549 cells and used guinea pig polyclonal sera or mouse monoclonal antibodies (mAbs) against either FHA or FIM to study the blocking effect on interactions of *B. pertussis* with host cells. We have shown that anti-FHA and anti-FIM antibodies can inhibit *B. pertussis* adhesion to host cells and reduce the bacterial growth. Furthermore, we showed that both anti-FIM and anti-FHA sera and mAbs induce *B. pertussis* aggregation and bacterial cell death, reducing bacterial growth. Inhibition of *B. pertussis* adhesion to A549 cells by antisera or mAbs also correlated with reduced production of inflammatory cytokines and increased A549 survival.

In summary, anti-FHA and anti-FIM antibodies were capable of sequestering *B. pertussis* in aggregates to kill the bacteria and prevent A549 cell inflammation, supporting the importance of humoral responses as a defense mechanism against *B. pertussis.*

## Material and Methods

### Reagents

Rabbit anti-human phospho p65 NF-κB, PathScan^®^ Signaling Nodes Multi-Target Sandwich ELISA, PathScan^®^ signaling NF-κB ELISA, and PathScan^®^ Sandwich ELISA Lysis Buffer were obtained from Cell Signaling Technology (Pickering, ON, Canada). Cy3-conjugated goat anti-mouse IgG was purchased from Invitrogen Canada (Burlington, ON, Canada). Bordet-Gengou blood agar plates were from BD Biosciences (Mississauga, ON, Canada). Syto9 and Propidium Iodide were from ThermoFisher (Waltham, MA). Wild-type *B. pertussis* (Tohama I) was propagated at Sanofi Pasteur (Toronto, ON, Canada). Human lung carcinoma epithelial A549 cell line was obtained from ATCC (Old Town Manassas, VA). For the cytokine detection (Eotaxin, Eotaxin-3, IL-10, IL-12/IL-23p40, IL-12p70, IL-13, IL-15, IL-16, IL-17A, IL-1α, IL-1β, IL-2, IL-4, IL-5, IL-6, IL-7, IL-8, IL-8 (HA), IL-21, IL-22, IL-23, IL-27, IL-31, IP-10, MCP-1, MCP-4, MDC, MIP-1α, MIP-1β, MIP-3α, TARC, TNF-α, TNF-β, VEGF-A, GM-CSF, and IFN-γ), V-PLEX Human Cytokine 36-Plex Kit (K15089D-2) was used for screening and only 20 selected cytokines were further tested on 20-plex cytokines (Meso Scale Discovery, Rockville, MD).

### Murine Monoclonal Antibodies

The monoclonal antibodies (mAbs) were generated through Hybridoma Technology by Envigo using purified in-house protein (against FIM, FHA, and PT) and are property of Sanofi Pasteur Limited. Anti-FHA mAbs (clones 2-14, 2-2, 1-7, 3-31, 3-35, 5-6, 28-1, 2-3, 1-14, 1-11, 32-1and 28-1) and anti-FIM mAbs (clones 1-1, 1-3, 1-7, C10, G10 and 1-10), and anti-PT mAbs (clones LP12 and PS21C) were first screened for their ability to inhibit *B. pertussis* growth. Selected anti-FHA (2-14, 1-7, 28-1, 11-1, 1-9, and 32-1) and anti-FIM (1-1, G10, 1-7, 1-10, and C10) mAbs were further investigated for co-culture studies using *B. pertussis* and A549 cells. Only anti-FHA (28-1) and FIM (1-10) were using for adhesion inhibition assay.

### Guinea Pig Immunization With aP Antigens

Twenty-five guinea pigs were immunized with single aP antigens (FIM or FHA) adjuvanted with AlPO_4_ or aP vaccine (which contains FIM, FHA, PT, PRN). Sera from guinea pigs immunized with diphtheria antigen were used as control in this study. All animals were housed in pathogen-free research vivarium at Sanofi Pasteur animal care facility. All procedures were compliant with Guidelines of the Canadian Council on Animal Care and recommendations of Helsinki protocol.

### A549 Cell and *Bordetella pertussis* Culture

*Bordetella pertussis* Tohama I strain was obtained from the collection of Microbiology Unit, Sanofi Pasteur, Canada. Human alveolar basal epithelial cells (A549) were cultured in Dulbecco’s Modified Eagle Medium (DMEM; Gibco, Gaithersburg, MD) supplemented with 10% fetal bovine serum (HyClone/Cytiva, Vancouver, British Columbia, Canada) and incubated at 37°C and 5% CO_2_. On the day of the assay, frozen vials of *B. pertussis* were thawed at room temperature. The bacteria were harvested by centrifugation at 10,000 x g for 3 min followed by re-suspension of the pellet in 1 ml of DPBS (Gibco), twice. For infection, 5 x 10^5^ A549 cells were co-cultured with 5.55 x 10^7^ *B. pertussis* at multiplicity of infection (MOI) of 111. Bacteria were first incubated with polyclonal sera or mAbs for 1 h before introduction to A549 cells. A549 cells and *B. pertussis* coculture was performed in 12-well plates. A549 cell morphological changes and bacterial aggregations were observed after 48 h of coculture by a bright-field compound microscope (Nikon) as previously described (22). After 72 h of incubation, the cell culture media from the co-culture plate was collected and centrifuged at 13,000 x g for 3 min. The cleared supernatants were stored at -20°C for cytokine and chemokine detections. The remaining pellet was resuspended in 1 ml of F11-medium. Bacterial concentration was determined by a spectrophotometer (SpectraMax, Molecular Devices, San Jose, CA) at OD_620 nm_. All bacterial clumps were broken up by vortexing and pipetting repeatedly. Dispersion of clumps was confirmed using light microcopy. Afterwards, bacteria were diluted 10^7^-fold by serial dilutions and 0.5 ml was plated on Bordet-Gengou agar and colonies counted after 5–7 days of culture at 37°C.

For adhesion assay, co-culture suspension was cultured on a sterile cover slip, then fixed with 4% paraformaldehyde (PFA) before immunofluorescence staining. To test the direct bactericidal effects of anti-pertussis antibodies, *B. pertussis* was grown in the presence of mAbs or anti-sera, in the absence of A549 cells, as mentioned above.

### A549 Cell Lysate Preparation for NF-κB Detection

After 48 h of co-culture of A549 cells with *B. pertussis*, culture supernatants were removed, and adherent cells washed three times with 1 ml of cold DPBS. Other controls including a positive control (LPS) and a negative control (resting cells) were also included. A549 cells were trypsinized and incubated with 0.5 ml ice-cold 1X cell lysis buffer with a protease inhibitor for 15 min on ice. Cytoplasmic fractions of cell lysates were obtained by centrifugation at 10,000 x *g* for 5 min at 4°C. The supernatant of cell lysates was collected for NF-κB detection and stored at –20°C as single-use aliquots.

### Cytoplasmic Phospho-p65 NF-κB Detection by ELISA

The cytoplasmic phospho- NF-κB p65 (Ser536) levels were determined as per manufacturer’s instructions (Cell Signaling Technology^®^). Briefly, 100 μl of each undiluted A549 cell lysate was added to the pre-coated plate and incubated overnight at 4°C. After four washes of the plate with 200 μl of 1X wash buffer, 100 μl of reconstituted detection antibody was added to each well. The plate was incubated at 37°C for 1 h. After washing, 100 μl of reconstituted HRP-linked secondary antibody was added to each well and the plates were incubated for 30 min at 37°C. After another four washes, 100 μl of TMB substrate was added to each well and the plates incubated at 37°C for 30 min. Reactions were stopped with 100 μl/well of maleic acid and the plates were read within 30 min by a spectrophotometer (SpectraMax) at 450 nm.

### Detection of Cytokines and Chemokines Using Meso Scale Discovery Technology

The assay for detection of cytokines, chemokines and other inflammatory mediators was performed as per the manufacturer’s recommendation (MSD). Briefly, 50 μl cell supernatant from 72h cell culture media and standards were added to MSD 20-plex precoated plates. The plate was incubated at room temperature for 2 h and washed 3 times with wash buffer to remove the unbound analytes. Twenty-five microliters of diluted SULFO-TAG–conjugated detection antibodies were added, and the plate was incubated for 2 h at room temperature. The plate was washed tree times, MSD “Read” buffer was added, and the plate was analyzed with an MSD instrument.

### Immunofluorescence Staining, Microscopy, ImageStream, and Flow Cytometry

After 48 h of co-culture of *B. pertussis* and anti-FIM or anti-FHA mAbs with A549 cells, the supernatant was removed, and adherent cells washed 3 times with 1 ml of DPBS. After blocking (4% bovine serum albumin (BSA) in 1X Tris-buffered saline (TBS)-Tween), adherent bacteria on A549 cells were stained with secondary goat anti-mouse Cy3-conjugated antibody to reveal the presence of anti-pertussis mAbs [control (Ctrl) anti-PRN (3-5), anti-FHA (28-1) anti-FIM (1-10)] on the bacteria. After washing, the cells were mounted with ProLong™ Gold Antifade Mountant (ThermoFisher) containing DAPI and observed by a fluorescent microscope (Zeiss Axio; Zeiss; Oberkochen, Germany).

Bacterial viability assessment was performed by staining a fraction of the suspension of mAb-treated bacteria collected after 48h culture. Bacteria were stained with 0.0125 µM of Syto9 and 7.5 µM of propidium iodide (PI) for 15 min. Fluorescently stained bacteria were analyzed with Flow cytometry (BD FACSCalibur, BD).

To investigate the correlation between bacterial death and aggregation, Syto9 or Propidium Iodide PI stained *B. pertussis* were analyzed on the Amnis^®^ ImageStream^®^X Mk II Imaging Flow Cytometer. Both dyes were excited with the 488 nm laser, and the Syto 9 or PI fluorescence was detected with either a 505–560 nm or a 595–642 nm band pass filter respectively at 60x magnification. The brightfield image and side scatter were also captured. Samples were analyzed using IDEAS software version 6.2.187.0. To analyze the bacteria, in focus events were gated using “Gradient RMS” values over 40, followed by Syto 9 and PI positive events with fluorescence intensities greater than 500 and 1000, respectively. Aggregated dead bacteria were defined as having large size *via* the brightfield image and/or having high mean pixel intensity of Syto9 fluorescence.

### Statistical Analysis

Data are shown as the mean ± SEM. Statistical comparisons were made using an unpaired Student’s t-test. Immunofluorescence analysis was performed using Image J software (National Institutes of Health). P values ≤ 0.05 were considered statistically significant.

## Results

### Interactions Between A549 Epithelial Cells and *Bordetella pertussis* Enhance Bacterial Growth Which Is Inhibited by Specific mAbs Against FIM and FHA

**Figure 1:**
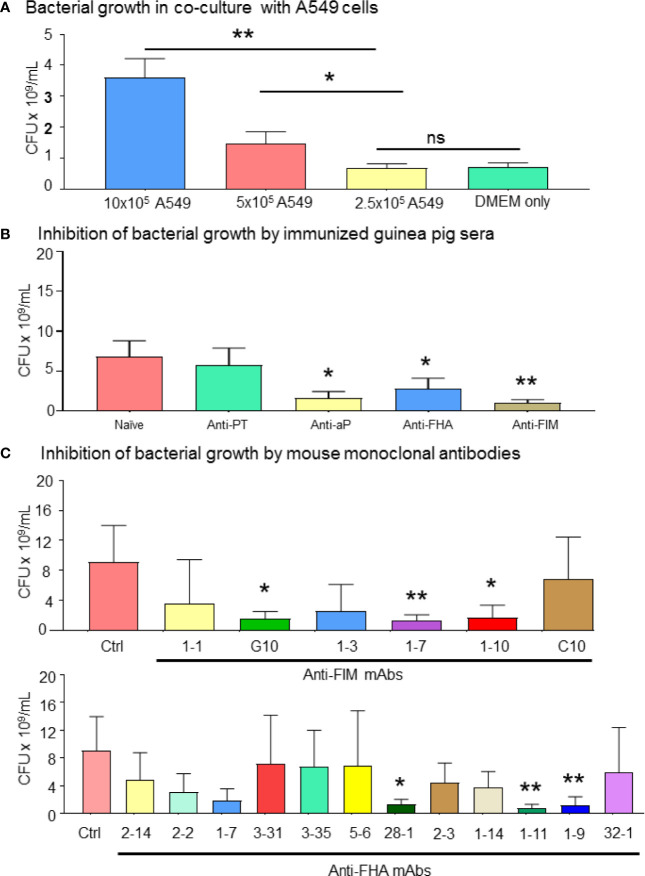
*Bordetella pertussis* interactions with A549 epithelial cells are essential for bacterial growth which is inhibited by specific mAbs. **(A)** *B. pertussis* growth significantly increased in a dose dependent manner in presence of 1 and 0.5 million of A549 cells. After 72 h co-culture of *B. pertussis* with A549 cells, extracellular bacterial concentration in the medium was determined. **(B, C)** Enumeration of live bacteria on Bordet-Gengou blood agar showed significant inhibition of *B. pertussis* growth by anti-sera **(B)** compared to naïve control sera. Mouse mAb targeting FIM (G10, 1-7, and 1-10) and FHA (28-1, 1-11, and 1-9) significantly inhibited bacterial growth compared to anti-RSV control mAb **(C)**. Unpaired Student’s *t*-test. Mean ± SEM. *p < 0.05, and **p < 0.01, n ≥ 4 experiments. ns, non significant.

During pertussis infection, common adhesion molecules (FIM and FHA) bind to their cognate ligands on epithelial cells, allowing the bacteria to escape the immune system and colonize the respiratory tract. In a co-culture system, we observed that bacterial growth was increased in the presence of A549 epithelial cells in a dose-dependent manner, compared to *B. pertussis* cultured alone in complete DMEM media (**Figure 1A**). Through this model, we were able to evaluate the inhibition of bacterial proliferation in the presence of guinea pig polyclonal sera and mouse monoclonal antibodies. The ability of antisera (polyclonal sera against either purified FIM or FHA antigen, or from immunization with aP vaccine) to reduce bacterial growth was first explored. To enumerate live bacteria, *B. pertussis* in the supernatants from 72 h co-cultures were plated on Bordet-Gengou agar. The results showed that the guinea pig antisera raised against aP vaccine, FHA or FIM purified antigens, significantly reduced the bacterial growth compared to naïve control sera or antisera raised against PT (**Figure 1B**). The reduction of bacterial growth in the presence of antibodies could also be achieved using anti-FHA (clones 28-1, 1-11, and 1-9) and anti-FIM (clones G10, 1-7, and 1-10) mAbs on bacterial growth (**Figure 1C**). The data suggested inhibition of *B. pertussis* growth by anti-FIM and anti-FHA antibodies through a mechanism that involves prevention of *B. pertussis* interactions with A549 cells.

### Polyclonal Antisera and Specific mAbs Induce *Bordetella pertussis* Aggregation and Improve A549 Cell Survival

**Figure 2:**
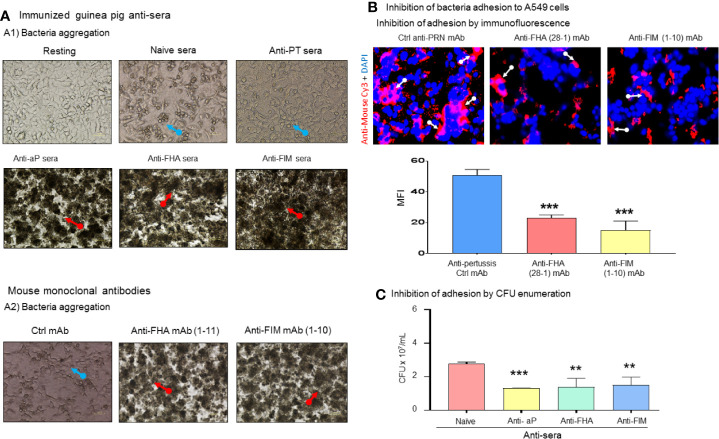
Antibodies against *B. pertussis* key adhesins induced bacterial aggregation and inhibit adhesion. **(A)** Naïve control sera did not protect infected A549 cells which exhibited cluster formation and cell death (blue arrows) compared to confluent resting cells. Naïve sera did not induce bacteria aggregation (A1). Antisera containing antibodies against aP vaccine or single immunogens, FHA, and FIM, induced bacterial aggregation (red arrows) in 100% of tested sera as certain anti-FHA and anti-FIM mAbs induced bacterial aggregation (A2). **(B)** Specific anti-pertussis antibodies inhibit bacterial adhesion. After 48 h of co-culture, anti-FIM and anti-FHA mAb significantly inhibited bacteria adhesion compared to control anti-PRN mAb (white arrow) to A549 cells which was confirmed using bacteria enumeration on Bordet-Gengou plate **(C)**. Mean ± SEM. ***p < 0.001 and **p < 0.01, n ≥ 3 experiments.

To explore the potential mechanism involved in bacterial growth inhibition, aggregation of *B. pertussis* in the presence of antibodies were investigated. Resting A549 cells reached 98%–100% confluency after 72 h of culture and formed a healthy monolayer. Infection of A549 cells by *B. pertussis* in the presence of naïve sera led to epithelial cell death, clump or cluster formation, and reduction in numbers of A549 cells (**Figure 2A**). Interestingly, antibodies against aP vaccine, anti-FHA, or anti-FIM sera induced bacterial aggregation after 48 h of incubation. We have shown that anti-PT sera induced weak and reversable aggregation in only half of the tested samples (data not shown). The anti-PT sera was used in this assay to compare the results with anti-FIM and anti-FHA sera. Anti- FHA mAbs (seven out of 12 clones) and all anti-FIM mAbs (six out of six clones) induced *B. pertussis* aggregation (data not shown).

The role of *B. pertussis* adhesins in binding to A549 cells was also evaluated in this study. After 48 h incubation of mAb-treated *B. pertussis* with A549 cells, an adhesion assay was performed. To visualize the interaction of bacteria with A549 cells, anti-FIM (1-10) and anti-FHA (28-1) mAbs bound to the *B. pertussis* surface were stained with Cy3-conjugated goat anti-mouse antibodies. Notably anti-FHA and anti-FIM reduced bacteria adhesion to A549 cells (**Figure 2B**) as revealed by fluorescence microscopy. Anti-PRN mAb used as the control as we have previously shown that this mAb does not induce aggregation. In addition to microscopy observations, we tested the ability of aggregation-inducing antibodies to prevent bacterial adhesion to A549 cells following 48 h of coculture. After removing unbound *B. pertussis* through washing, aP vaccine, anti-FHA, and anti-FIM sera, which induced bacteria aggregation, were shown to significantly reduce CFU count of adherent bacteria (**Figure 2C**).

### Aggregation by Anti-Pertussis Antibodies Induces Direct Bactericidal Effect on *Bordetella pertussis*, and Restores A549 Cell Survival

**Figure 3:**
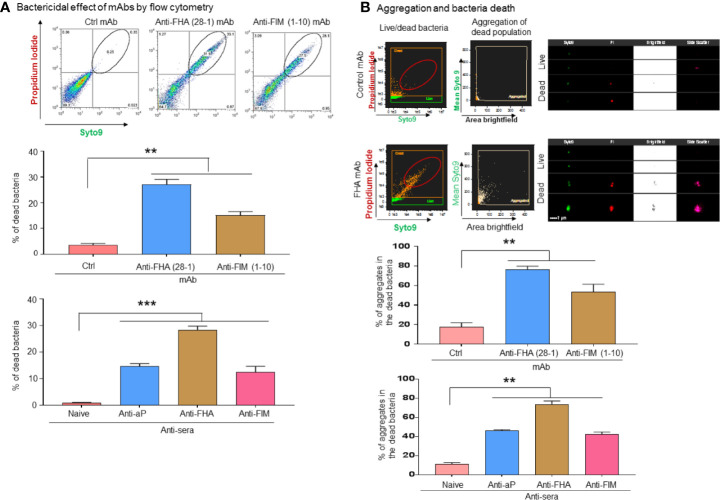
*B. pertussis* sequestration by aggregation inducing antibodies mediated direct bactericidal effects. *B. pertussis* was incubated for 48 h with anti-pertussis antibodies without A549 cells to test the direct bactericidal effect of anti-FIM and anti-FHA antibodies. Flow cytometry-based analysis of bacteria death shows that anti-pertussis antibodies against FIM and FHA exhibit direct bactericidal effect **(A)**. Furthermore, analysis by imaging flow cytometry showed that majority of dead bacteria are within the aggregates **(B)**. Unpaired Student’s *t*-test. Mean ± SEM. **p < 0.01 and ***p < 0.001, n ≥ 3 experiments.

A flow cytometry-based approach was developed and used to further investigate the bactericidal properties of anti-pertussis mAb. For this purpose, anti-pertussis antibody-treated bacteria were cultured in DMEM for 48 h without A549 cells. Staining of live/dead bacteria indicated that the bacterial death occurs in the presence of anti-FIM (1-10) or anti-FHA (28-1) antibodies alone (**Figure 3A**). Complementary analysis using a flow imaging system (ImageStream) showed a similar pattern to the traditional flow cytometry (**Figures 3A, B**). In addition, the ImageStream system was used to further analyze aggregation within the dead bacteria population using high magnification (60X). In this study, aggregation was defined as events with large size by brightfield and/or with high mean Syto 9 fluorescence, an indication that the bacteria are tightly compacted (**Figure 3B**, center plot). Treatment of *B. pertussis* with FIM (1-10) or FHA (28-1) antibodies resulted in the formation of large aggregates in the dead bacteria population as compare to the control. The potential of anti-pertussis antibodies to prevent attachment or adhesion (which is a key step for infection) to epithelial cells or to kill *B. pertussis* in the aggregates may represent an interesting mechanism of action of mAbs on the pathogen.

### Antibodies That Mediate *B. pertussis* Aggregation Restores A549 Cell Survival and Alters Cytokine/Chemokine Secretions

**Figure 4:**
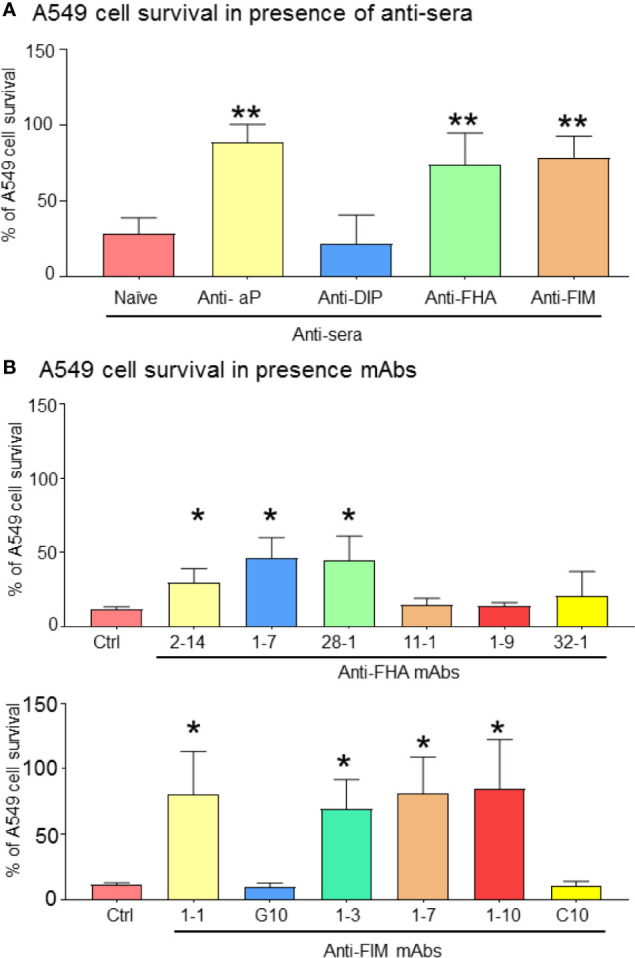
Bacteria aggregation and inhibition of adhesion improve A549 cell survival. After 72 h of infection, remaining adherent A549 cells were stained with Neutral red for cell viability/proliferation. Anti-FIM and anti-FHA anti-sera, which induced B. pertussis aggregation have a beneficial impact on A549 cell survival **(A)**. This observation was reproducible by certain specific anti-FIM and anti-FHA mAbs **(B)**. Unpaired Student’s t-test. Mean ± SEM. *p < 0.05 and **p < 0.01. n ≥ 3 experiments.

To evaluate the effects of anti-pertussis antibodies on A549 cells, a neutral red viability assay was conducted in order to assess cell survival in the presence of *B. pertussis*. Neither naïve sera or anti-diphtheria toxin (DIP) control sera protected A549 cells from *B. pertussis*, induced A549 cell cluster formation and cell death (**Figure 4A**). In contrast, antisera containing antibodies against aP vaccine, FHA, or FIM antigens significantly improved A549 cell survival (**Figure 4A**). This was also observed in the presence of three anti-FHA mAbs (clones 2-14, 1-7, and 28-1) and three anti-FIM mAbs (clones 1-1, 1-3, 1-7, and 1-10), which significantly prevented A549 cell death.

**Figure 5:**
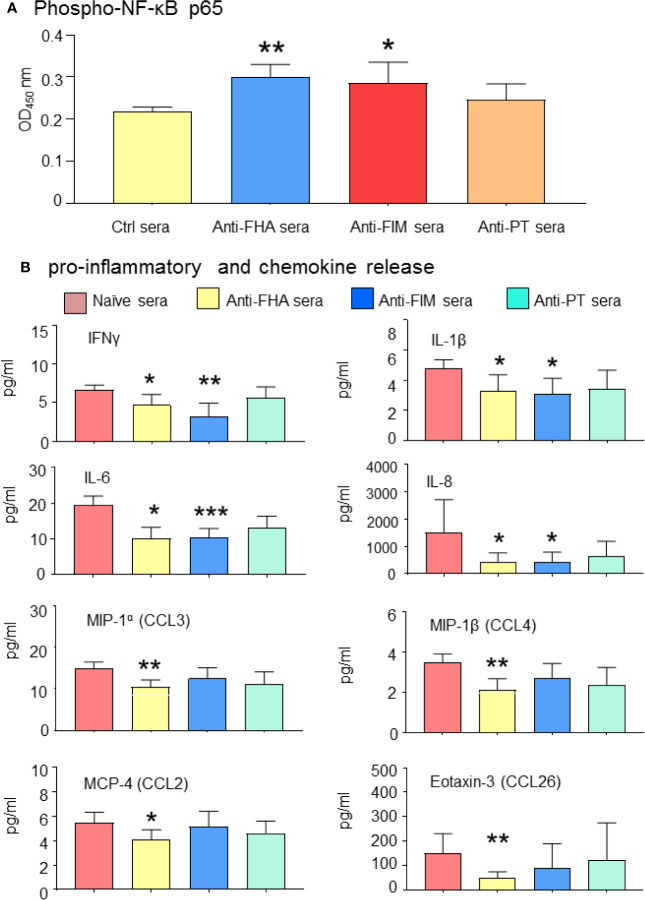
Antibodies mediating *B. pertussis* aggregation reduced A549 pro-inflammatory signaling. **(A)** Anti-FHA and anti-FIM polyclonal sera reduced NF-κB activation and A549 cell inflammatory response. Cytoplasmic NF-κB levels remains higher in presence of anti-FIM and anti-FHA sera compared to control sera. **(B)** Complementary analysis of pro-inflammatory cytokines and chemokines released in the culture supernatant in response to *B. pertussis* infection were significantly reduced by anti-FIM and anti-FHA compared to naive sera. Unpaired Student’s *t*-test. Mean ± SEM. *p < 0.05, **p < 0.01, ***p < 0.001 n ≥ 3 experiments.

To evaluate the impact of the inhibition of *B. pertussis* interaction with A549 cells by anti-FIM and anti-FHA sera, pro-inflammatory signaling and cytokine secretions were investigated in the co-cultured A549 cells. Specifically, this work leveraged NF-κB, a downstream target mediating inflammation which has previously been shown to be activated upon by binding of *B. pertussis* adhesins to Toll-like receptors TLR2 and TLR4 (23). Translocation of NF-κB from the cytoplasm to the nucleus initiates transcription of various cytokine genes. In this study, the level of cytoplasmic phosphor-p65 of NF-κB was enhanced in the presence of anti-FIM or anti-FHA sera, suggesting less translocation of NF-κB to the host cell nucleus and reduced transcription of pro-inflammatory genes. As expected, in the presence of anti-PT sera, the level of cytoplasmic phosphor-p65 was similar to control sera (**Figure 5A**).

Activation of NF-κB is a prerequisite for pro-inflammatory cytokine secretions; therefore, the cytokine profile of A549 infected cells in the presence of different antisera was further investigated. Thirty-six cytokines were first screened by a multiplex panel and then narrowed down to 20 cytokines and chemokines of interest. The level of several pro-inflammatory cytokines (IFNγ, IL-1β, IL-6, and IL-8) and chemokines (MIP-1α chemokine (C-C motif) ligand (CCL3), MIP-1β (CCL4), MCP-4 (CCL2), and Eotaxin-3 (CCL26) were significantly reduced in the presence of anti-FHA or anti-FIM sera (**Figure 5B**). These results indicate that *B. pertussis* aggregation prevents interaction between bacteria and A549 cells, thus improving epithelial cell survival and preventing proinflammatory response.

## Discussion

Pertussis continues to be a major public health concern especially in infants despite high coverage of vaccines (7). Recent findings have shown the urgent need for pertussis vaccines, particularly in the prenatal stage of life, to reduce severe pertussis in newborns (24). For this particular reason, the advisory committee on immunization practices of Centers for Disease Control and Prevention recommended immunization of pregnant women with acellular pertussis vaccine to protect their infants against pertussis *via* transplacental antibody transfer (8, 25). Antibodies play a major role in the protection of newborns from severe pertussis, however the mechanisms by which anti-pertussis antibodies exert direct bactericidal effects to control epithelial infection has remained elusive (26).

From this study, one possible mechanism of action for antibodies against certain *B. pertussis* adhesins is the induction of bacterial aggregation to sequester and kill the bacteria. We have performed multiple assays to obtain mechanistic insight into the bactericidal properties of anti-pertussis antibodies and their protective effects on A549 cells. We found that in the presence of *B. pertussis*, A549 epithelial cells release biological factors supporting bacterial growth. By sequestering *B. pertussis* into aggregates, anti-FIM and anti-FHA antibodies inhibit bacteria interactions with A549 cells, a prerequisite for enhanced bacterial growth in our co-culture system.

The observation that *B. pertussis* experiences more rapid growth in the presence of A549 cells prompted us to establish a cellular model for proper investigation on the bactericidal properties of vaccine-induced antibodies. We hypothesize that during infection, interaction of *B. pertussis* with epithelial cells mediate release of variety of factors from infected A549 cells which facilitate bacteria growth through a direct pathogen-host cell interaction. This is in agreement with a previous study showing that *B. pertussis* interactions with respiratory epithelial ciliated cells led to five-fold increase in bacterial density within 24 h (27). When *B. pertussis* interacts with epithelial cells through its major adhesins, it may induce signaling for bacterial replication. Using filtered media from the co-culture system, enhanced bacterial growth was observed; albeit further study is required to identify and investigate any possible secreted biological factor(s) and/or property of this ‘conditioned’ A549 cell media that may impact *B. pertussis* growth. Some factors such as MgSO_4_ and glutamate have been previously reported to affect *B. pertussis* growth (28, 29). For instance, conditions that restrict the availability of glutamate, a key essential amino acid, have been showed to reduce expression of virulence factors and adhesins, and entering stationary phase of *B. pertussis*. Since *B. pertussis* does not utilize sugars, but rather rely on glutamate, deprivation of this essential nutrient has been shown to induce autoaggregation (28). The fact that FHA and FIM are highly expressed on the pathogen at all stages of bacterial growth makes these antigens effectively targetable by antibodies. In fact, when *B. pertussis* is co-cultured with A549 in the presence of anti-FHA and anti-FIM mAbs or polyclonal sera, inhibition of bacterial adhesion/interactions prevented biological factor release. Inhibition of bacteria interactions with A549 cells by anti-FHA and FIM antibodies could explain the reduction of *B. pertussis* growth, however this does not occur without aggregation which sequesters the pathogen.

To increase pertussis vaccine efficacy, antigens that induce generation of bactericidal antibodies have been highly recommended. Of particular interest, engineering vaccines that promote antibodies with opsonophagocytic and/or bactericidal activity may improve efficacy (30). There are discrepancies between the existing studies investigating opsonophagocytic and bactericidal activities induced by aP vaccines (31, 32). In a study by Lesne and colleagues, contribution of PRN to generate complement-mediated bactericidal antibodies has been reported while anti-FIM, anti-FHA, and anti-PT antibodies against pertussis were not involved (31). However, some controversies remain as other studies showed that levels of anti-PT and FIM antibodies directly correlated with the protection in humans (32, 33). In our study all the animals immunized with FHA or FIM immunogens developed antibodies that induce *B. pertussis* aggregation. Aggregation can be visualized after 24 h and aggregates grew over the time. It is a dynamic process in which the aggregates become larger over time after 48 h. Bacterial death within the aggregates was demonstrated by examining at the population of aggregated *B. pertussis* stained by Syto 9 and propidium iodide live/dead markers. Aggregation blocks pathogen-host cell interactions, which may in turn disrupt some factors that are needed for bacterial proliferation (e.g. host signals that trigger bacterial proliferation or growth, or factors that may be derived from other bacterial interactions). It is also known that *B. pertussis* is very sensitive to temperature and nutrients (e.g. MgSO_4_ and glutamate) for expression of key virulent factors. It is possible that the contact inhibition between neighboring bacteria may trigger a decline in cell cycle or even more cell death. Taken together, microbial pathogens must sense local conditions, including space to grow, nutrient availability, and adjust their growth state accordingly to survive in a host environment. Therefore, anti-FHA and anti-FIM antibodies may block the biosynthesis of necessary virulence factors associated with cell division.

In agreement with bacterial aggregation and growth inhibition by anti-FIM and anti-FHA mAbs, survival of A549 cells was significantly improved. Stimulation of inflammation was also prevented as key anti-inflammatory cytokines and chemokines were reduced in the presence of anti-FIM and anti-FHA antibodies (Scheme 1). By sequestering *B. pertussis* in aggregates, anti-pertussis antibodies can reduce bacterial growth and prevent A549 cell inflammation, demonstrating their potential for development of *in vitro* potency assays, supporting initiatives to reduce animal-based testing.

## Data Availability Statement

The raw data supporting the conclusions of this article will be made available by the authors, without undue reservation.

## Author Contributions

IY designed and performed all the experiments, analyzed and interpreted the data, and prepared and revised the manuscript. AM conducted several cell-based assays and prepared the data. LH and CC conducted flow cytometry experiments and prepared the manuscript. JS and BG provided guidance for study design and data interpretation. AA supervised the study, interpreted the data, and revised the manuscript. All authors contributed to the article and approved the submitted version.

## Conflict of Interest

All authors are employees of Sanofi Pasteur.
